# Vitamin D and Cardiovascular Health: A Narrative Review of Risk Reduction Evidence

**DOI:** 10.3390/nu17132102

**Published:** 2025-06-25

**Authors:** William B. Grant, Barbara J. Boucher, Richard Z. Cheng, Pawel Pludowski, Sunil J. Wimalawansa

**Affiliations:** 1 Sunlight, Nutrition, and Health Research Center, 1745 Pacific Ave., Ste. 504, San Francisco, CA 94109, USA; 2 The Blizard Institute, Barts and The London School of Medicine and Dentistry, Queen Mary University of London, London E1 2AT, UK; bboucher@doctors.org.uk; 3 Orthomolecular Medicine News Service, Columbia, SC 29212, USA; richzc@gmail.com; 4 Low Carb Medicine Alliance, Shanghai 201613, China; 5 Department of Clinical Biochemistry, The Children’s Memorial Health Institute, 04-730 Warsaw, Poland; p.pludowski@ipczd.pl; 6 Endocrinology & Human Nutrition, Department of Medicine, Cardiometabolic & Endocrine Institute, North Brunswick, NJ 08902, USA; suniljw@hotmail.com

**Keywords:** cardiovascular disease, causality, heart, Mendelian randomization, meta-analysis, prospective cohort studies, randomized controlled trials, stroke, vitamin D, 25-hydroxyvitamin D

## Abstract

The role of vitamin D in reducing cardiovascular disease (CVD) risk remains debated despite growing evidence. Prospective observational studies consistently show that low serum 25-hydroxyvitamin D [25(OH)D] concentrations (below 40–50 nmol/L [16–20 ng/mL]) are associated with the highest risk of CVD incidence. In addition, a large prospective observational study found that serum 25(OH)D concentration was inversely correlated with CVD mortality rate to over 100 nmol/L. Randomized controlled trials have not generally demonstrated benefit due to faulty study designs, such as enrolling participants with baseline 25(OH)D levels > 50 nmol/L. However, a major trial found that 60,000 IU/month of vitamin D_3_ supplementation reduced the risk of major cardiovascular events for participants with predicted 25(OH)D concentrations ≥ 50 nmol/L or taking statins or CV drugs by ~13 to ~17%. In addition, vitamin D supplementation studies have found modest reductions in several CVD risk factors. Other observational studies of vitamin D supplementation have reported reduced CVD risks (e.g., ischemic heart disease, hypertension, and myocardial infarction). Temporal ecological studies further support this relationship, revealing that CVD incidence rates are lowest in summer and CVD mortality rates are significantly higher in late winter—when 25(OH)D concentrations are lowest—compared to late summer. A previously reported analysis using eight of Hill’s criteria for causality in a biological system further strengthens the biological plausibility of vitamin D’s role in CVD risk reduction. Its role in modulating inflammation and oxidative stress, improving endothelial function, and reducing several cardiometabolic risk factors supports its inclusion as part of a comprehensive, multi-modal approach to cardiovascular health. Therefore, vitamin D should be considered an integral component in the prevention and management of CVD. Preferably, it should be used in combination with other nutritional supplements, a heart-healthy diet, and prescription medications to reduce the risk of CVD incidence. People should consider vitamin D_3_ supplementation with at least 2000 IU/day (50 mcg/day) (more for those who are obese) when sun exposure is insufficient to maintain serum 25(OH)D concentrations above 75 nmol/L. To reduce CVD mortality rates, higher doses to achieve higher 25(OH)D concentrations might be warranted.

## 1. Introduction

Cardiovascular disease (CVD) is a leading cause of death. In the U.S. in 2022, CVD accounted for 941,652 deaths (39.5% of the total) [1]. Of those deaths, 39.5% were from coronary heart disease (CHD); 17.1% from stroke; 17.0% from other causes; 14.5% from hypertensive diseases; 9.3% from heart failure; 9.3%; and 2.6% from diseases of the arteries. The global prevalence of CVD for 2025 was estimated at 598 million, and global CVD deaths at 20.5 million [2]. Thus, finding ways to reduce the risk of CVD is warranted.

There has been a long-standing debate regarding the role of vitamin D in reducing the risk of CVD. In 1981, Scragg proposed that variations in vitamin D produced by solar UVB caused some seasonal variation in CVD incidence rates in addition to the effects of temperature and respiratory illness [3]. In a community-based study published in 1990, he reported that myocardial infarction (MI) incidence rates were correlated inversely with 25-hydroxyvitamin D [25(OH)D] concentrations [4] in 179 MI patients and 179 controls in that New Zealand study. The relative risk of MI for 25(OH)D at or above the median (32 nmol/L) (to convert to ng/mL, divided by 2.5) was 0.43 (95% confidence interval [CI], 0.27–0.68) vs. below the median. Despite his four-decade-long following of this topic, he recently concluded that vitamin D does not prevent CVD [5]. This was because the recent Endocrine Society analysis of 14 randomized controlled trials (RCTs) of vitamin D supplementation compared to placebo found no beneficial effect on the incidence of CVD [6,7].

The evidence regarding vitamin D’s role in CVD risk, however, comes from several types of studies other than RCTs: temporal ecological studies, case–control studies, prospective cohort studies, and observational studies of vitamin D supplementation, as well as mechanistic and Mendelian randomization analysis (MRA) studies. While RCTs are used as the standard approach for determining the efficacy of pharmacological agents, they are unsuitable for assessing the effectiveness of micronutrients. At the same time, observational studies are most appropriate for micronutrients, such as vitamin D, for assessing the risk reduction in many diseases. A recent review examined the observational study and RCT evidence regarding vitamin D and the ten leading causes of death in the U.S. [8]. It found limited support from RCTs but strong support from observational studies for eight of the well-recognized leading causes of death: heart disease, cancer, COVID-19, stroke, chronic lower respiratory diseases, Alzheimer’s disease (AD), diabetes mellitus, and kidney disease. Unfortunately, the MRA evidence for heart disease was found to be inaccurate (discussed later in this review), which led us to draft this review regarding vitamin D and CVD.

RCTs are used for medications to evaluate the use of drugs to prevent and treat diseases. These drugs are not found in nature, whereas vitamin D is. Additionally, pharmacological agents have narrow dose–response curves [9]. In contrast, nutrients are threshold agents and have broader and often S-shaped dose–response curves [10].

There are several reasons for the failure of RCTs in evaluating micronutrients, including vitamin D. Key reasons include enrolling subjects with relatively high serum 25(OH)D concentrations (i.e., vitamin D sufficient), giving those in the treatment arm vitamin D doses too low to normalize vitamin D status, trials being of too short duration, and permitting those in the control arm to take vitamin D and/or giving them low vitamin D doses for ethical reasons [10]. In addition, both groups are exposed to uncontrolled ambient UVB radiation. Furthermore, most studies evaluate the results using intention-to-treat analysis rather than analyzing the results by achieving serum 25(OH)D concentrations. This narrative review, therefore, aims to evaluate the evidence critically regarding the role of vitamin D in reducing the risk of CVDs to conclude its causality.

## 2. Materials and Methods

Peer-reviewed Journal literature searches were conducted in Google Scholar and PubMed.gov. Vitamin D emerges as a compelling micronutrient in the realm of cardiovascular disease prevention, offering a promising area for further exploration despite the challenges posed by traditional randomized controlled trials (RCTs). The nuanced interplay between serum 25-hydroxyvitamin D [25(OH)D] concentrations and cardiovascular outcomes has been elucidated through various observational methodologies, emphasizing the necessity of tailored research approaches for definitive conclusions.

This report includes data obtained by searches using the terms case–control, cohort, ecological, mechanisms, Mendelian randomization, meta-analysis; observational, RCT, review, supplementation, diseases, atherosclerosis, cardiovascular disease, CHD, myocardial infarction, stroke, serum 25-hydroxyvitamin D concentration, and vitamin D derived from MeSH and EMTREE thesaurus terms and used both individually and in combination.

## 3. Results

### 3.1. Prospective Observational Studies

Prospective observational studies have examined the relationship between baseline serum 25(OH)D concentration and risk of various CVDs. The first such study was published in 1990 regarding MI incidence [4]. The next study regarding CVD incidence was not published until 2008 [11]. It is based on data from the Framingham Offspring Study. This study involved 1739 participants with a mean age of 59 years, followed by a mean time of 5.4 years, of whom 120 developed a first CVD event. The adjusted hazard ratio (aHR) for 25(OH)D <15 ng/mL vs. >15 ng/mL was 1.62 (95% CI, 1.11–2.36), while that for 25(OH)D <10 ng/mL vs. >10 ng/mL was 1.80 (95% CI, 1.05–3.08) and for participants with hypertension, the aHR was 2.13 (95% CI, 1.30–3.48).

Many subsequent prospective cohort studies have found similar results. Table 1 gives the findings from studies that reported results between 1990 and 2010, plus two later studies. The studies included were found from searches of Google Scholar and PubMed.gov, as well as those included in a meta-analysis by Grandi [12]. Three studies included in ‘Grandi’s meta-analysis were omitted: Melamed et al. [13] since it was a cross-sectional study; Kilkkinen [14] because it had a mean follow-up period of 27 years; and Bolland et al. [15] due to a small number of participants. The purpose of this table is to show that it was well understood that the risk of CVD was related to low 25(OH)D concentrations before most of the RCTs regarding vitamin D and risk of CVD were designed and conducted. As can be seen, there were significant relative risks for serum 25(OH)D concentration below 25–37 nmol/L compared with higher than 37 or 75 nmol/L. The risk was greater for the mortality rate than for the incidence rate. The mean age of the participants also affected the risk of CVD mortality. For participants with a mean age of 44 years, RR = 1.39 (1.02–1.89) [16] while for a mean age of 75 years, RR = 2.64 (1.14–4.79) [17]. The Anderson et al. study [18] shows that the risk for CVD incidence for 25(OH)D = 40–75 vs. >75 nmol/L is one-third as large as for <35 vs. >75 nmol/L, supporting the idea that participants in RCTs to evaluate the effect of vitamin D supplementation should have 25(OH)D concentrations below 40–50 nmol/L.

Prospective cohort studies and mechanistic analyses together paint a vivid picture of vitamin D’s potential in modulating cardiovascular health, specifically by influencing systemic risk factors such as hypertension and myocardial infarctions. The dynamic relationship between ambient UVB radiation exposure and seasonal shifts in CVD incidence further imbues this narrative with biological plausibility, underscoring the need for innovative trial designs that account for environmental and demographic variabilities. Through these lenses, a critical perspective emerges on the optimal use of vitamin D supplementation and its thresholds to achieve meaningful reductions in mortality and morbidity from CVD.

An interesting study from Turkey examined serum 25(OH)D concentrations in obese patients regarding the severity of CHD [25]. The study involved 120 obese patients with a mean age of 62 ± 11 years who underwent coronary angiography between May 2012 and June 2023. They calculated the SYNTAX scores for each patient. The SYNTAX score makes a detailed analysis of the coronary arteries, which can be used to identify the safest revascularization modality. The SYNTAX Score II 2020 was found to predict prognosis after percutaneous coronary intervention and coronary artery bypass grafting [26]. It predicted mortality well and showed some utility in predicting which of the two modalities would have a better outcome. Table 2 in [25] gives the multivariable analysis for age, sex, hypertension, diabetes mellitus, smoking, low-density lipoprotein-cholesterol (LDL-C), and 25(OH)D concentration. Only 25(OH)D had a significant OR (0.81 [95% CI, 0.74–0.88, *p* < 0.001]). Figure 1 in [25] then compared 25(OH)D concentrations with SYNTAX scores. The SYNTAX scores dropped from >28 with values of <30 nmol/L to <22 for values of 25(OH)D >58 nmol/L. In addition, a mean serum 25(OH)D concentration of 34.7 nmol/L predicted high SYNTAX scores with 81% sensitivity and 80.6% specificity [19]. This study provides additional evidence that serum 25(OH)D concentration is consistently found to be inversely correlated with CVD severity.

A relatively unrecognized problem with prospective cohort studies is “regression dilution” due to variable changes during post-baseline follow-up. The classic paper regarding this effect was published in 1999 [27]. It analyzed serial measurements of systolic blood pressure (SBP), diastolic BP, and blood cholesterol amongst participants in the Framingham Study. The risk ratio for systolic BP for the first and fifth quantiles of vitamin D status changed from 1.46 at baseline to 1.29 at six years, 1.21 at 16 years, and 1.13 at 26 years, and similar changes were seen for diastolic BP and blood cholesterol.

**Figure 1:**
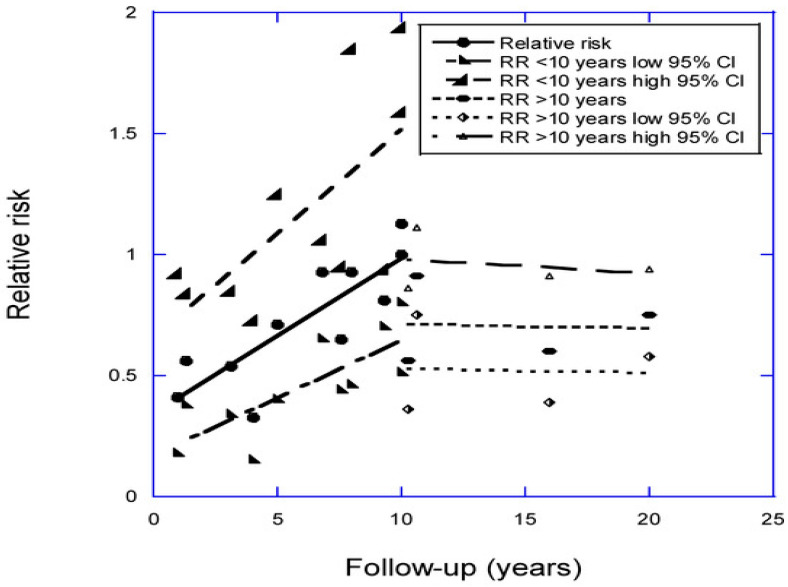
The plot of relative risk for stroke versus years of follow-up with respect to high vs. low 25(OH)D concentration, with regression fits found for studies of less than 10 years and for those carried out over more than 10 years; 95% CI, 95% confidence interval [28]. This figure is from an open-access article distributed under the terms and conditions of the Creative Commons Attribution (CC BY) license (https://creativecommons.org/licenses/by/4.0/, accessed on 10 December 2024).

The effect of the follow-up period for prospective cohort studies of stroke and major adverse cardiovascular events (MACE) was examined in a 2024 review [28]. There were 11 stroke studies with good data for follow-up periods of 1.0 to 10 years. Most studies compared results for serum 25(OH)D values of >50 nmol/L vs. <50 nmol/L. A plot of the relative risk of stroke vs. years of follow-up is shown in Figure 1 from that review [28].

The regression for the best fit for the relative risk (RR) data for these studies was 0.34 + (0.065 *×* follow-up [years]), *r* = 0.84, adjusted *r*^2^ = 0.67, *p* < 0.001). For one year, the RR = 0.41 (95% CI, 0.22–0.61). The mean RR reported in the meta-analysis for high vs. low 25(OH)D concentration for 21 studies by Su et al. [29] was 0.78 (95% CI, 0.70–0.86). The mean RR reported in the meta-analysis for low vs. high 25(OH)D concentration from 19 studies by Xiong et al. [30] was 1.45 (95% CI, 1.20–1.74) or, for high vs. low 25(OH)D concentration, 0.69 (95% CI, 0.57–0.83). Thus, the estimate of the RR for stroke adjusted for the follow-up period and using the value for the shortest follow-up period is about 70–90% larger than if not adjusted for the follow-up period.

For MACE, seven studies in the meta-analysis by Zhang et al., with mean follow-up periods < 9 years, were used [31]. The regression fit to the data for low vs. high 25(OH)D (generally <50 vs. >75 mol/L) was RR = 0.61 + (0.055 *×* follow-up [years]), *r* = 0.81, adjusted *r*^2^ = 0.59, *p* = 0.03). For one year, the RR = 0.67 (95% CI, 0.48–0.91). The overall RR for low vs. high 25(OH)D for eight studies in Zhang et al. was RR = 1.33 (95% CI, 1.18–1.49) or, for high vs. low 25(OH)D concentration, RR = 0.75 (95% CI, 0.67–0.85). [Note, the follow-up RR after adjustment for the overall follow-up period is 12% higher than shown in the forest plot analysis]. This result is important when evaluating the role of vitamin D in the risk of CVD using Hill’s criteria for causality in a biological system [32].

A meta-analysis of 15 prospective studies of CVD mortality and four studies of sudden cardiac deaths was used to calculate the dose–response analysis between serum 25(OH)D concentration and mortality rate [33]. The forest plot ‘analysis’s overall HR for the lowest vs. highest serum 25(OH)D concentration category was 1.75 (95% CI, 1.49–2.06). With 100 nmol/L as the reference, the HRs (95% CI) for 25(OH)D of 60, 40, 20, and 12 nmol/L were 1.10 (0.96–1.27), 1.28 (1.11–1.47), 1.55 (1.27–1.910, and 1.73 (1.34–2.27), respectively.

The mean follow-up period after measuring the variables affect the overall relative risk as well as the dose–response curves between serum 25(OH)D concentration and health outcome. That was demonstrated in the analysis of the follow-up period for serum 25(OH)D concentration and colorectal cancer (CRC) incidence. A meta-analysis involving 17 prospective cohort studies developed 25(OH)D concentration dose-CRC incidence relationships [34]. For each 25 nmol/L increment in circulating 25(OH)D, CRC risk was 19% lower in women (RR = 0.81 [95% CI, 0.75–0.87]) and 7% lower in men (RR = 0.93 [95% CI, 0.86–1.00). The RR from each study used in that meta-analysis was plotted vs. the mean follow-up period, as shown in Figure 1 in [35]. Therefore, the RR for a zero follow-up period should be approximately 0.75 for men and 0.77 for women. The regression fit to the data for men is OR = 0.74 + 0.031 *×* years, *r* = 0.79, adjusted *r^2^* = 0.59, *p* = 0.002; the regression fit to the data for women is OR = 0.77 + 0.008 *×* years, *r* = 0.25, adjusted *r*^2^ = 0, *p* = 0.42.

Additional observational evidence that vitamin D is associated with the risk of stroke comes from other studies, including one in India [36], where serum 25(OH)D concentrations were measured for 86 ischemic stroke patients within 24 h of admission to a hospital and compared to stroke severity using the National Institutes of Health Stroke Scale (NIHSS). Figure 1 in that article shows a linear fit to the data, starting with an NIHSS score near 12 for 18 nmol/L and dropping to 6 near 70 nmol/L [36]. The Pearson correlation coefficient, *r*, for the NIHSS score vs. 25(OH)D concentration was −0.41, *r*^2^ = 0.17, *p* < 0.001. These data suggest that patients with known CVD might well benefit from higher vitamin D status.

A meta-analysis was performed using individual participant data to analyze the association between standardized serum 25(OH)D and CVD mortality using observational data from six European studies [37] found that the HRs for CVD deaths adjusted for age, sex, season of blood draw and body mass index at baseline were 1.69 (95% CI, 1.47–1.95), 1.93 (95% CI, 1.60–2.22), and 3.10 (95% CI, 1.78–5.41) for 25(OH)D values from 40 to 50 nmol/L, 30–40 nmol/L, and <30 nmol/L, respectively, and this finding is likely to relate to the previously reported increases in BP seen with lower serum 25(OH)D concentrations.

In another large study, the dose–response curve for serum 25(OH)D concentration-CVD mortality rate was analyzed in 37,079 patients with CVD from the UK Biobank study [38]. In the fully adjusted model with 25(OH)D <25 nmol/L set to 1.00; HR for 25.0–49.9 nmol/L = 0.79 (95% CI, 0.71–0.89); for 50.0–74.9 nmol/L, = 0.71 (95% CI, 0.63–0.81), and for >75 nmol/L, HR = 0.59 (95% CI, 0.49–0.70). The range of 25(OH)D concentration >75 nmol/L extended to 150 nmol/L, where the RR was 0.36 (95% CI, 0.23–0.52). These data suggest that patients with known CVD might usefully be supplemented with vitamin D.

High blood pressure, or hypertension, is an important risk factor for CVD. An analysis of blood pressure data on the risk of CVD was conducted in Japan [39]. The study involved 4231 participants with a mean age of 65 ± 10 years, 91.5% of whom were hypertensives. Each participant measured BP in the morning for 14 consecutive days in each of the four seasons. The measure extracted from these data was the average real variability for all three-day intervals (there are 12 three-day intervals in the 14 days). In the fully adjusted model, average real variability per the standard deviation in winter had the highest HR for CVD, 1.44 (95% CI, 1.17–1.77, *p* < 0.001). This finding strengthens the association between hypertension and total CVD risk, and since hypertensive risks are increased in association with lower vitamin D status, it supports the likelihood that better vitamin D status should reduce CVD risks.

### 3.2. Temporal Ecological Studies

As mentioned in the introduction, Scragg was the first to suggest that seasonal variations in solar UVB doses and serum 25(OH)D concentrations contributed to the seasonal variations in CVD mortality rates, highest in winter and lowest in summer [3]. The seasonality of CV risk factors (CVRFs) based on data from 230,000 participants in 15 countries was published in 2014 [40]. Scragg was one of the co-authors. Minor seasonal variations in BMI, waist circumference, systolic and diastolic BP, and cholesterol were found. It suggested that seasonal variations in temperature, serum 25(OH)D concentrations, air pollution, diet, and physical activity could explain the seasonal variations in CVRFs. For 25(OH)D concentrations, it referenced a 2007 analysis of data on CVRFs from the Third National Health and Nutrition Examination Survey [41]; that article noted that 25(OH)D concentrations were lower for those with hypertension, diabetes mellitus, obesity, and high serum triglyceride (TG) levels.

That study was quickly followed by a 19-country study regarding the role of solar radiation on seasonal variation in mortality rates and various disorders [42]. In ten northern latitude countries (seven in Europe, Canada, the U.S., and Taiwan), the second winter peak CVD mortality rate was in February, while the summertime minimum mortality rate was in September. In three southern latitude countries, Australia, Chile, and South Africa, the wintertime maximum mortality rate was in July and or August, while the summertime mortality rate minimum was in February. Those authors suggested that the most likely cause of the seasonal variations in CVD mortality rates was seasonal temperature variation, but that other factors, including dietary habits, sun exposure, serum 25(OH)D concentrations, and human parasitic and infectious agents, could also play roles.

According to a large observational study of seasonal variations in serum 25(OH)D concentrations in the UK of 7437 White 45-year-old participants, the mean values in summer and winter were 70 and 35 nmol/L, respectively [43]. That decrease of 35 nmol/L could translate into a 5.6-mmHg increase in BP for those with vitamin D deficiency, and, judging by the effect of pharmacological BP lowering on the risk of CVD as determined in an individual participant-level data meta-analysis [44], results in a clinically significant increase in complications and deaths [See Table 3 in [44] reporting that for each 5-mmHg reduction in systolic BP, HR = 0.90 (95% CI, 0.88–0.92) for MACEs and = 0.87 (95% CI, 0.84–0.91) for stroke]. Thus, the rise in BP with winter reductions in vitamin D status could account for some of the increase in CVD risks seen in the winter [45].

Seasonal variations in low 25(OH)D concentrations have been reported in two U.S. clinical studies. The first one reported data from 3.4 million measurements made on samples sent to the Mayo Clinic (Rochester, MN, USA) from all over the U.S. from late 2006 to the end of 2011 [46] and found that the percentage of values below 30 nmol/L (severe vitamin D deficiency [less than 12 ng/mL]) increased from 1.5% in summer to 9% in winter, while the percentage of values from 30 to 60 nmol/L increased from 17% to 28%. The second study was based on 3.8 million measurements in the Quest Diagnostics database from across the U.S. for 2007–2009 [47] and found that the percentage of 25(OH)D results below 50 nmol/L increased from 13% in summer to 31% in winter. Furthermore, an analysis of data from the UK Biobank found that the proportion of participants with 25(OH)D concentration <25 nmol/L increased from 3.5% in summer to 24–30% in winter [48].

Two authors of the present review examined the seasonal variation in CVD mortality rates in two articles [49,50]. The 2017 article [49] discussed the effects of seasonal variations in solar UVB doses, serum 25(OH)D concentrations, respiratory tract infections, gene expression, temperature, and air pollution on seasonal variations in total and CVD mortality rates in the U.S., finding that seasonal variations in solar UVB and serum 25(OH)D concentrations had the most significant impact. In addition, it mentioned the Christmas-New Year’s holiday peak outlined by Phillips et al. in 2010 [51].

The 2022 article [50] examined the roles of atmospheric temperature and humidity, solar UVB/vitamin D, and UV/nitric oxide (NO). Based on a study from the Global Burden of Disease Study [52], it was estimated that low temperatures could account for 0.5% of CVD deaths at 15° latitude, rising to 2.5% at latitude 40°. The work by Weller et al. regarding UV increases in serum NO concentration and reductions in BP [53] discussed the roles of NO and vitamin D produced by sun exposure, arguing that NO had a more important role in affecting BP than vitamin D [54].

Weller dismissed vitamin D’s effect by citing a 2014 article showing that oral vitamin D did not affect BP [55]. However, he overlooked two studies reporting that vitamin D supplementation reduced BP. One study was an RCT for subjects with low 25(OH)D concentrations [56]. Another was an observational study of participants with high 25(OH)D concentrations [57]. Both studies are discussed later in this review. However, Weller did not provide strong evidence that UV effectively lowered BP through increasing serum NO concentrations. It is now understood that blue light and perhaps red and near-infrared radiation are probably more important than UV in raising serum NO concentrations [58].

Unfortunately, the evidence cited in the 2022 article [50] for UVB/vitamin D, reducing the risk of CVD was not very strong either, since the most substantial evidence was from two MR studies [59,60] that were later retracted [61,62]. Overlooked in that article, however, was a 2019 meta-analysis of the association between serum 25(OH)D concentration and CVD incidence and mortality rates from prospective cohort studies by Gholami et al. [63]. It found that for low vs. high serum 25(OH)D concentrations, the CVD incidence RR from six studies = 1.18 (95% CI, 1.00–1.39), and for CVD mortality rate from 19 studies, the overall RR = 1.54 (95% CI, 1.29–1.84). This analysis shows that the effect of vitamin D on CVD is much stronger for mortality rates than incidence rates. Gholami et al. [63], also investigating whether there was a publication bias in the prospective studies, found none according to Egger’s test (*p* = 0.76). They did find asymmetry in the funnel plot, which they attributed to small-study effects, but excluding the three small studies did not change the results significantly.

A 2025 research article by Oskarsson et al. [64], based on data from the BiomarCaRE project [65], compared CVD outcomes concerning serum 25(OH)D concentrations and their associated seasonal variation. The authors argued that the January peak in CVD mortality rates preceded the 25(OH)D concentration trough by two to three months, violating one of several Hill’s criteria for causality in a biological system [32]. The authors rejected ‘Scragg’s hypothesis that short-term reductions in 25(OH)D concentrations lead to seasonal increases in CVD outcomes [3]. The January peak is the well-known Christmas–New Year’s holiday peak due to population mixing in indoor situations and over-indulgence during celebrations [51], while population mixing in winter increases the risk of influenza and other viral respiratory diseases—a known trigger for CVD mortality [66].

In Oskarsson et al.’s data, there is a second peak of CVD mortality rate ([33 ± 4 deaths]/10,000 person-years [PYs]) in April, the third month of the lowest 25(OH)D concentrations [64]. The lowest CVD mortality rate ([25 ± 3 deaths]/10,000 PYs) occurred in September, after the third month of high summertime 25(OH)D concentrations. The highest to lowest CVD mortality rate ratio is significantly higher: (by 8 ± 5 deaths)/10,000 PYs, an increase of 30 ± 20%. There is also evidence of lowering CVD mortality from May onwards (i.e., the summer effect) [64]. Thus, short-term reductions in 25(OH)D can affect seasonal increases in CVD mortality rates, as proposed by Scragg [3]. Note, too, that July and August CVD incidence rates were significantly lower than for all other months: for July, RR = 0.91 (95% CI, 0.83–0.99), and for August, RR = 0.89 (95% CI, 0.80–0.96). Thus, higher 25(OH)D concentrations in summer can be associated with reduced CVD incidence rates in Europe. There were no significantly increased CVD incidence rates in winter. Thus, these results are similar to the findings from prospective cohort studies in that the effect of 25(OH)D on the CVD mortality rate was more substantial than the effect on the CVD incidence rate, as shown in Table 1.

### 3.3. Vitamin D Supplementation Observational Studies

An analysis of atherosclerotic CVD (ACVD) rates determined from the UK Biobank dataset [67] was published shortly prior to the publication of Oskarsson et al. [64]. Figure 2 in that article [67] shows that the risk of ACVD, HR = 1.07 (95% CI, 1.03–1.12) at 33 nmol/L and increases to 1.23 (95% CI, 1.18–1.31) at 10 nmol/L and low 25(OH)D concentrations are more likely to be present in winter than summer. In addition, they compared data from 17,801 vitamin D users vs. 306,985 non-vitamin D users. As shown in Table 3 in [67], for atherosclerotic cardiovascular disease, the fully adjusted data for all identified confounding factors showed HR = 0.94 (95% CI, 0.90–0.98, *p* = 0.02). For ischemic heart disease (IHD), the fully adjusted model HR = 0.90 (95% CI, 0.86–0.96, *p* = 0.002).

A vitamin D supplementation study of myocardial infarction (MI) risk was published in 2021 [68]. It was a retrospective, observational, nested case–control study of 20,025 patients with 25(OH)D concentrations <50 nmol/L who received care at the U.S. Veterans Health Administration from 1999 to 2018. Those patients who achieved >75 nmol/L through vitamin D supplementation had a significantly lower risk of MI than untreated patients who remained <50 nmol/L (HR = 0.73 [95% CI, 0.55–0.96, *p* = 0.02]) [67,68]. Those patients who achieved 50–75 nmol/L through vitamin D supplementation had a significantly lower risk of MI than untreated patients who remained <50 nmol/L (HR = 0.65 [95% CI, 0.49–0.85, *p* = 0.002]). The reason for a lower HR for 50–75 nmol/L than for>75 nmol/L is not known. Experimental studies have demonstrated that vitamin D inhibits the transformation of macrophages to foam cells, increases cholesterol efflux in macrophages, improves endothelial nitric oxide formation, promotes vascular repair, decreases thrombogenicity as well as inflammation, and, in addition, reduces their secretion of destructive MMPs. All these mechanisms may play a role in providing a protective effect against the atherothrombotic process, such as MI [69,70,71].

### 3.4. Randomized Controlled Trials

Many vitamin D RCTs have not found that vitamin D supplementation reduces the risk of CVD. Likely reasons include that few participants with 25(OH)D concentrations below 50 nmol/L were included, that participants in the control groups were either given low vitamin D doses or permitted to take vitamin D supplements, and that the results were not evaluated for achieved 25(OH)D concentrations [72]. The 2019 Barbarawi et al. [39] meta-analysis included overall RRs for four CVD outcomes. For vitamin D supplementation vs. placebo for MACE based on ten studies, the overall RR was 1.00 (95% CI, 0.95–1.05); for CVD death based on ten studies, the overall RR was 0.98 (95% CI, 0.90–1.07); for myocardial infarction (MI) based on 18 studies, the overall RR was 1.00 (95% CI, 0.91–1.08); for cerebrovascular accident, the overall RR was 1.06 (95% CI, 0.98–1.15).

A meta-analysis of CV events from vitamin D RCTs with a low risk of bias was made for the 2024 Endocrine Society Clinical Practice Guidelines on Vitamin D [6]. Data for those studies are given in Table 2. Mean baseline 25(OH)D concentrations were between 49 and 79 nmol/L, as shown in the meta-analysis on p. 114 of the supplementary file to that report [6], and the overall RR was 0.99 (95% CI, 0.91–1.07).

Interestingly, one of the RCTs, the D-Health trial from Australia [75], found one significant reduction and two marginal (and non-significant) reductions for some MACEs. The HR for vitamin D treatment vs. placebo was for MACE, 0.91 (95% CI, 0.91–1.01), for MI, 0.81 (95% CI,0.67–0.98), and for coronary revascularization, 0.89 (95% CI, 0.78–1.10). This is the only major RCT that has found vitamin D supplementation reduced MACE risks. Figure 3 in [75] shows that predicted baseline 25(OH)D concentration ≥ 50 nmol/L, use of statins, and use of CV drugs predicted independent beneficial effects of vitamin D in reducing the incidence of MACE: HR = 0.87 (95% CI, 0.76–0.98); 0.83 (0.71–0.97); and 0.84 (95% CI, 0.74–97), respectively. It would be interesting to learn whether reanalyses of other major vitamin D RCTs would show similar effects. This finding implies that anyone being treated with drugs to reduce the risk of CVD should also be taking vitamin D_3_ supplements of at least 2000 IU/day.

Observational studies have found that the 25(OH)D concentrations associated with the highest risk of CVD are those below 37 nmol/L, as shown in Table 1, so RCTs could not be expected to find that vitamin D supplementation would reduce the risk of CVD or MI unless the participants all had 25(OH)D concentrations below 37 nmol/L. Thus, the fact that RCTs have not demonstrated that vitamin D supplementation reduces the risk of CVD or MI is not evidence that vitamin D does not reduce the risk of CVD or MI; nor is it evidence of reverse causality, as proposed by Autier [84,85]. It merely reflects that the RCTs have not been adequately designed, conducted, or analyzed, as discussed recently [72,86]. It also indicates that vitamin D RCTs should be guided by prospective cohort studies and designed to be capable of evaluating relevant findings.

### 3.5. Vitamin D Supplementation Studies Regarding CVD Risk Factors

There are many important risk factors for CVD. A 2022 review provided a global ranking of the attributable burden of CVD due to selected modifiable risk factors [87]. The rank of those that vitamin D might affect are, from Table 1 in [87]: 1, high SBP; 3, high LDL-C; 6, fasting glucose; 7, high BMI: 8, kidney function. In addition, effects on high-density-lipoprotein-cholesterol (HDL-C) and TGs were sought. HDL-C and triglicerides are considered possible risk-modifying factors for CVD, both individually and in the ratio TG/HDL-C [88]. A search was made for vitamin D supplementation studies reporting beneficial effects for these risk factors. Those with beneficial findings are listed in Table 3. Significant reductions for higher achieved 25(OH)D concentrations were found for aortic BP, fasting glucose level (FBG), glycated hemoglobin (HbAic), homeostasis model assessment of insulin resistance (HOMA-IR), LDL-C, pulse wave velocity, peak reservoir pressure, SBP, and TG, and increases in HDL-C. Note that mean baseline serum 25(OH)D concentrations were at or below 41 nmol/L for all but two of the studies. In one of those, BP was lowered by increasing serum 25(OH)D concentrations above 100 nmol/L [57]. Such studies strengthen the case for the role of vitamin D in reducing the risk of CVD.

### 3.6. Mechanisms Where Vitamin D Reduces the Risk of CVD

The mechanisms whereby vitamin D reduces the risk of CVD are generally well known. These include reductions in BP (by suppressing renin secretion [94]) and reductions in inflammation, a significant factor in the progression of atheromatous arterial disease. By suppression of pro- and stimulation of anti-inflammatory cytokine secretion [95], the risks of arterial plaque instability, which leads to local clot formation and resultant acute events, are reduced. The plaque instability aggravation by invading macrophages is due to their secretion of the matrix metalloproteinase 2/9 enzymes, which are suppressed by vitamin D, which should reduce the risks of acute CVD events [96].

A 2021 review listed eight major hypothetical mechanisms underlying the association between vitamin D and CVD: cardiac hypertrophy, diabetes mellitus, endothelial dysfunction, inflammation, myocardial fibrosis, oxidative stress, renin–angiotensin–aldosterone system activation, and systemic hypertension [97]. This understanding of the mechanisms of worsening CVD in vitamin D deficiency satisfies the plausibility clause of ‘Hill’s criteria for causality [32].

Additional reviews of the role of vitamin D in stroke prevention [98] report a role for reduction in the risks of metabolic syndrome abnormalities by vitamin D, since those abnormalities contribute to CVD risks [99] because vitamin D is known to reduce insulin resistance, the risks of Type 2 diabetes in prediabetes, and the risks of metabolic syndrome [100], further explaining the role of deficiency in the pathogenesis of CVD and cerebrovascular diseases [101]. The fact that obesity directly reduces serum 25(OH)D through reductions in hepatic 25-hydroxylation of vitamin D will tend to increase CVD risks. However, this element of CVD risk can be overcome by ensuring a 1.5-to-3-fold increase in vitamin D provision in overweight/obese people [102,103].

### 3.7. Mendelian Randomization Analysis Studies

It was brought to our attention by Professor George Davey Smith (MRC Integrative Epidemiology Unit, University of Bristol, Bristol, UK) that an article based on non-linear MRA [60] that we had quoted in our recent review [8] as reporting significant CVD risk reductions with higher vitamin D status was retracted in February 2025 [62]. Another similar non-linear MRA regarding vitamin D and CVD [59] was also retracted in 1 December 2023 [61] and three of his recent papers now outline the problems caused by current non-linear MRA methodology when used for GWAS data in general. For example, non-linear MRA data analyses produced findings suggesting that higher serum LDL-C significantly reduced CVD risks when, in fact, higher LDL-C is one of the well-known risk factors for CVD [104,105,106].

### 3.8. Hill’s Criteria for Causality

Medical systems use RCTs to examine the efficacy of pharmaceutical drugs. Such drugs are rarely found in the natural environment, so they need to be tested for efficacy and adverse health effects. The medical system has adopted RCTs as being required for vitamin D in preventing and treating disease, and has requested RCTs for vitamin D. However, Hill’s criteria for causality [32] are a scientific way to assess causality and can include RCTs as one of the criteria. Hill’s criteria have been used to evaluate whether vitamin D can be linked causally to reduced risk for various diseases, including cancer [107] and dementia [108]. Hill’s criteria were evaluated for vitamin D and CVD in 2014 [109] and in a similar review in 2018 [110]. The only criterion not considered satisfied was experimental verification, such as by a randomized controlled trial. Table 4 lists the criteria appropriate for vitamin D and how they have been satisfied. As can be seen, the criteria are generally satisfied except for RCTs, as already discussed, which we have already shown to be due, in the main, to their unsuitable design.

After the submission of this manuscript, we found the recent review of the vitamin D and CVD hypothesis by Kiely et al. [114]. This report includes analyses of the evidence for vitamin D and MI/CHD, stroke, and both CVD and all-cause mortality, and on vitamin D using Hill’s criteria for causality. There is general agreement between the two analyses. However, they consider consistency to be best satisfied for three outcomes, temporality to be best satisfied for three outcomes, while experimental verification was not satisfied for CVD mortality or stroke since it was untested, doubtful for MI/CHD, but satisfied for all-cause mortality. They also noted that vitamin D RCTs conducted to date have largely failed to test the underlying fundamental hypothesis. Thus, those analyses do not detract from the recognition that correction of vitamin D deficiency could benefit any CVD disorder arising from or exacerbated by that deficiency.

## 4. Discussion

After 44 years of research, the mechanisms by which vitamin D can reduce CVD risks are very well understood. However, the clinical evidence for those benefits remains inconclusive, largely due to poor RCT design. However, few RCTs carried out have been suitably designed to be able to investigate the effect of vitamin D supplementation on CVD risk factors on participants with 25(OH)D concentrations below 50 nmol/L. On the other hand, two observational studies of vitamin D supplementation have shown significant reductions in the risk of MI, atherosclerotic cardiovascular disease, and IHD rates with higher serum 25(OH)D values over time. In addition, the vitamin D-CVD association satisfies Hill’s criteria for causality in a biological system.

The Italian National Institute for Cardiovascular Research has recently considered the current evidence to be supportive of an association between low 25(OH)D concentrations and increased CV mortality [115]. As a result, it recommends a personalized approach for vitamin D supplementation for CV health. A review conducted in the United Arab Emirates also concluded that to promote cardiovascular health and prevent the worsening of cardiovascular risk factors, high doses of vitamin D (above 2000 IU/day) may be required, and doses less than this are likely to be insufficient in those with deficiency [116].

In vitamin D RCTs, achieving proper study groups, especially the control group, with low 25(OH)D concentrations (with low exposure to sunlight, dietary sources of vitamin D, over-the-counter supplements, etc.) is nearly impossible. In addition, attempting to conduct vitamin D clinical studies when participants with serum 25(OH)D concentration below 30 nmol/L in the control group are not provided with some vitamin D (such as 400 IU/day) often raises ethical concerns [117]. This challenge is minimized by conducting community-based prospective/ecological studies. Thus, the results of these two vitamin D supplementation studies [68] may be considered proxies for vitamin D randomized controlled trials. In addition, such studies would provide further data for testing Hill’s experimental verification criterion.

There are some guidelines for conducting an RCT that could allow a better evaluation of the role of vitamin D in the risk of CVD [10]. First, it should be acknowledged that the guidelines for RCTs for pharmaceutical drugs, when used for vitamin D, violate one of the fundamental assumptions of those guidelines: i.e., that the control group should not be taking any of the drugs being tested, since everyone has some vitamin D and giving the control group even small amounts of vitamin D dose can increase serum 25(OH)D concentrations enough to reduce RCT validity, as discussed in two recent reviews [72,86].

Robert Heaney outlined guidelines for RCTs of nutrients in 2014 [118]. As applied to vitamin D, those guidelines included measuring serum 25(OH)D concentrations of prospective participants and including only those with low concentrations, using findings from observational studies to estimate the achieved 25(OH)D concentration required for relevant health-risk reduction; giving treatment arm participants vitamin D doses that achieve the desired concentrations; measuring achieved 25(OH)D concentrations after a few months and adjusting the vitamin D doses as needed at intervals during the trial, and, analyzing trial results in terms of achieved 25(OH)D concentration, and NOT by intention to treat [119].

One of the reasons for using achieved 25(OH)D concentrations is that people have different changes in serum 25(OH)D concentration due to differences in absorption from the gastrointestinal tract and, in particular, due to variations in adiposity [102,103] and in vitamin D’s genetic pathways; one study, for example, has found that genetic variations can result in ±20% differences in achieved 25(OH)D concentrations with vitamin D supplementation [120].

Two trials provide useful examples of how to conduct vitamin D RCTs. One was a stratified randomized field trial involving vitamin D screening and treatment conducted in Iran [121]. It involved 900 pregnant women receiving prenatal care at two hospitals. Mean baseline serum 25(OH)D concentrations at each hospital were 28 (interquartile range, 18, 40) nmol/L. Subjects at one hospital were not supplemented with vitamin D. At the second hospital, women with deficiency [25(OH)D, 25 to 50 nmol/L] and severe [25(OH)D, <25 nmol/L] deficiency were randomly divided into four subgroups and received vitamin D_3_ designed to raise serum 25(OH)D to >50 nmol/L until delivery. Follow-up health care screening showed that supplementation reduced the risk of preeclampsia by 60% (OR, 0.40 [95% CI, 0.30–0.60]), gestational diabetes mellitus by 50% (OR, 0.50 [95% CI, 0.34–0.88]), and preterm delivery by 40% (OR, 0.60 [95% CI, 0.40–0.80]).

The second trial involved vitamin D supplementation for adults with prediabetes. This was the Vitamin D and Type 2 Diabetes D2d trial conducted by Pittas et al. [122]. It involved 2423 participants (1211 in the vitamin D group and 1212 in the placebo group). Those in the vitamin D group were given 4000 IU/d vitamin D_3_. The mean baseline 25(OH)D concentration was 70 nmol/L. The trial ran for 2.5 years. When the results were analyzed by intention to treat, HR = 0.88 (0.95% CI, 0.75–1.04; *p* = 0.12). However, in an article published in 2020 [123], the results were re-analyzed according to the 25(OH)D concentration achieved.

The HRs for diabetes among participants treated with vitamin D who maintained intra-trial 25(OH)D concentrations of 100–124 or 125 nmol/L and above were 0.48 (95% CI, 0.29–0.80 carlve3) and 0.29 (95% CI, 0.17–0.50), respectively, compared with those who maintained a concentration of 50–74 nmol/L. The HR for diabetes for an increase of 25 nmol/L in mean intra-trial 25(OH)D concentration was 0.75 (95% CI 0.68–0.82) among those assigned to vitamin D. There are several reasons why increases in serum 25(OH)D concentration with vitamin D supplementation can differ such including degrees of obesity and genetic variation in the vitamin D metabolic pathway as shown in Tables 2 and 3 in Ref. [120]. However, as shown in Ref. [123], adjusting the HR models for geographical site, BMI at baseline, race, sex, age, physical activity at baseline, and statin usage had little effect on the calculated HRs. The authors, therefore, recommend conducting additional, well-designed observational clinical studies to evaluate vitamin D’s role in reducing CVD risks. Furthermore, suppose further vitamin D RCTs are to be performed for CVD. In that case, their design must be carefully organized to be suitable for this nutrient, and if any further actions of vitamin D are suggested to reduce CVD risk, their mechanisms of action must be fully investigated.

The global prevalence of prediabetes in 2021 was estimated based on impaired insulin tolerance (IGT) data from 43 countries and impaired fasting glucose (IFG) data from 40 countries [124]. Based on IGT, it was 9.1% (464 million), while based on IFG, it was 5.8% (298 million). The prevalence rate based on IGT increases linearly from 5% in those aged 20–24 years to 17% for those aged 75–79 years. The prevalence rate based on IFG rises from 3.5% for those aged 20–24 years to 7% for those aged 50–59 years and remains about the same for higher age groups. The association between prediabetes and CVD was estimated from prospective cohort studies with a median follow-up of 9.5 years [125]. In a 2020 analysis, the composite RR for CVD with prediabetes was 1.15 (95% CI, 1.11–1.18) [126].

For CHD, the overall RR for prediabetes was 1.16 (95% CI, 1.11–1.21); for stroke, the overall RR for prediabetes was 1.14 (95% CI, 1.08–1.20). However, given the linear increase in prevalence of prediabetes with age, based on IGT rates, the RR for older people may be as much as twice those values. Also, the fact that long follow-up periods give rise to regression dilution [27], could also mean that the actual risk of CVD associated with prediabetes is higher than currently thought. Since raising serum 25(OH)D concentration to above 125 nmol/L with 4000 IU/day vitamin D_3_ would reduce the conversion of prediabetes to diabetes mellitus [123], it should also reduce the risk of CVD [127].

An analysis of 57 articles with 4.5 million persons with type 2 diabetes mellitus (T2DM) globally in 2007–2017 at a mean age of 64 ± 4 years for a mean duration of 10.4 ± 3.5 years determined the association of CVD with T2DM [128]. CVD affected 32.2% overall (53 studies, N = 4,289,140); 29.1% had atherosclerosis (4 studies, N = 1153), 21.2% had CHD (42 articles, N = 3,833,200), 14.9% heart failure (14 studies, N = 601,154), 14.6% angina (4 studies, N = 354,743), 10.0% myocardial infarction (13 studies, N = 3,518,833) and 7.6% stroke (39 studies, N = 3,901,505). CVD was the cause of death in 9.9% of T2DM patients (representing 50.3% of all deaths). Thus, prediabetes patients could significantly reduce their risk of CVD by supplementing with 4000 IU/d vitamin D_3_ to achieve serum 25(OH)D concentrations >125 nmol/L.

The adverse effects of vitamin D are extremely rare. The main concern is hypercalcemia and associated signs and symptoms, only seen when non-obese people have taken >30,000 IU/day for many months; the diagnosis depends on hypercalcemia-related side effects and hypercalciuria [129]. It is noted that in patients with granulomatous disease, such as sarcoidosis or tuberculosis, and tumors such as lymphomas, hypercalcemia occurs as a result of the activity of ectopic 25(OH)D-1-hydroxylase (CYP27B1) expressed in macrophages or tumor cells and the formation of excessive amounts of 1,25-(OH)_2_D [130].

## 5. Conclusions

A large body of research has found that vitamin D status is inversely associated with the risk of CVD. Vitamin D’s effect on CVD mortality rate is more important than its role in CVD incidence. Temporal ecological studies find that seasonal variations in solar UVB affect the risk of CVD incidence and mortality rates; prospective observational studies find that serum 25(OH)D concentrations are correlated inversely with the risk of CVD and MI, and observational studies show that vitamin D supplementation reduces BP, MI, ACHV, and IHD. Several mechanisms are known that help to explain how vitamin D reduces CVD risks. RCTs have not, to date, been designed to evaluate the findings from observational studies that very low 25(OH)D concentrations are strongly associated with the risk of CVD. Thus, it cannot be claimed that the lack of positive findings from RCTs demonstrates that vitamin D does not reduce the risk of CV. In contrast, the vitamin D-CVD hypothesis does satisfy Hill’s criteria for causality in a biological system.

Vitamin D supplementation might, therefore, be recommended to reduce the risk of CVD. For instance, serum 25(OH)D concentrations might be raised above 75 nmol/L in northern mid-to-upper latitude countries from the beginning of October through mid-June by giving a minimum of 2000 IU/day (50 micrograms/day) of vitamin D_3_ and 1.5-to-3 times increase in this dose for those who are overweight or obese). To reduce CVD mortality rates, serum 25(OH)D concentrations might be raised above 100–125 nmol/L with 5000 IU/day or more vitamin D_3_, depending on their body weight [131]. Doing so could also reduce the risk of other diseases, as outlined in our recent review [8]. Measurement of achieved 25(OH)D concentrations for confirmation of the adequacy of supplementation would also be helpful due to individual differences in vitamin D supplementation response.

## Figures and Tables

**Table 1:** Representative prospective observational studies of cardiovascular disease, myocardial infarction, stroke incidence, and/or mortality rates concerning baseline serum 25(OH)D concentrations published between 1990 and 2010, plus a few later studies.

Reference	Population Cases, Controls Mean BMI (kg/m^2^)	Outcome	Mean Follow-Up Period (Years)	Mean Age (Years)	25(OH)D (nmol/L)	RR (95% CI)
Scragg, 1990 [4]	26, 25	MI inc	0	53	≥32 vs. <32	0.43 (0.27–0.89)
Wang, 2008 [11]	28 ± 5	CVD inc	5.4	59 ± 9	<37 vs. ≥37	1.62 (1.11–2.36)
					<25 vs. ≥37	1.80 (1.05–3.08)
		CVD inc *			<37 vs. ≥37	2.13 (1.30–3.48)
Giovannucci, 2008 [19]	26 ± 3	MI inc	10	64 ± 9	<37 vs. ≥75	2.42 (1.53–3.84)
Dobnig, 2008 [20]	27 ± 3	CVD mor	7.7	63 ± 9	19 vs. 71 (md)	2.08 (1.60–2.70)
					33 vs. 71 (md)	1.53 (1.17–2.01)
Pilz, 2009 [21]	27 ± 5	CVD mor	6.2	70 ± 7	31 vs. 58 + 79 (md)	5.33 (1.97–14.45)
Anderson, 2010 [18]		CAD/MI	1.3 ± 1.2	55 ± 21	<38 vs. >75	1.45 (*p* = 0.003)
					40–75 vs. >75	1.15 (*p* = 0.09)
		HF			<38 vs. >75	2.10 (*p* < 0.001)
					40–75 vs. >75	1.31 (*p* = 0.005)
		Stroke			<38 vs. >75	1.78 (*p* = 0.004)
					40–75 vs. >75	1.31 (*p* = 0.01)
		CVD inc			<38 vs. >75	1.79 (1.53–2.10)
					40–75 vs. >75	1.22 (1.08–1.38)
Ginde, 2009 [22]	27	CVD mor	7.3	74	<25 vs. ≥100	2.36 (1.17–4.75)
Semba, 2010 [17]	27 ± 3	CVD mor	6.5	~75 ± 3	<26 vs. >66	2.64 (1.14–4.79)
Grandi, 2010 [12]		CVD inc	MA, 4 studies		Low vs. high	1.54 (1.22–1.96)
		CVD mor	MA, 5 studies		Low vs. high	1.83 (1.19–2.80)
Schöttker, 2013 [16]	28 ± 4	CVD mor	9.5	62 ± 7	<30 vs. >50	1.39 (1.02–1.89)
Wang, 2021 [23]	29	Stroke inc	5.5	47	<50 vs. 50–125	1.77 (1.27–3.07)
Jayedi, 2023 [24]		CVD mor **	MA, 5 studies		<25 vs. ≥50	1.70 (1.17–1.37)
		CVD inc **	MA, 6 studies		<25 vs. ≥50	1.27 (1.34–2.06)

(*) for participants with hypertension; (**), for participants with prediabetes or type 2 diabetes mellitus; CAD, coronary artery disease; CVD, cardiovascular disease; HF, heart failure; hypertension; inc incidence; MA, meta-analysis; md, median; MI, myocardial infarction; mor, mortality; RR, relative risk.

**Table 2:** Data from RCTs with vitamin D supplementation regarding CV events with low risk of bias reported on p. 114 in Shah et al. [6].

Author	Baseline 25(OH)D (nmol/L)	BMI	Age (Years)	Vitamin D_3_ Dose (IU) Treatment, Placebo	Duration (Years)	n	Country	%
Manson, 2019 [73]	F, 79 M, 70	28 ± 6	67 ± 7	2000/d 0	5.3	25,871	USA	29.21
Scragg, 2017 [74]	64 ± 24	28.4	50–84	100 k/mo, 0	3.3	5110	NZ	23.28
Thompson, 2023 [75]	77?		69 ± 6	60 k/mo	5	21,310	Australia	16.02
Virtanen, 2022 [76]	75 ± 18	27 ± 3	68 ± 5	1600/d 3200/d 0	5	2495	Finland	3.90
Joseph, 2023 [77]	64 ± 6		F > 55 M > 50		4.6	2835, 25,670	Developing countries	2.16
Baron, 2015 [78]	61 ± 20	29 ± 5	58 ± 7	1000/day	3–5	2259	USA	1.33
Hansen, 2015 [79]	<75	31 ± 7	60 ± 6	50 k/2 wk 800/15 d 0	1	229	USA	0.64
Aloia, 2018 [80]	55 ± 17	30 ± 4	68 ± 4	To achieve >75 nmol/L 0	3	260	USA	0.35
Witham, 2013 [81]	?		64 ± 10 68 ± 11	300 k/2 mo 0	0.5	75	UK	0.35
Lee, 2016 [82]	49	27 ± 5		4000/d 2000/d 0	1	290	UK	0.20
Epstein, 2012 [83]	64 ± 3	27 ± 5	71 ± 6	1000/d 400/d 0	1	305	UK	0.05

BMI, body mass index; d, day; k, kilo; mo, month; n, number of participants; wk, week.

**Table 3:** Findings from studies reporting beneficial effects of vitamin D supplementation on modifiable risk factors for CVD.

**Author**	**Population**	**BMI, Age (Years)**	**Baseline** **25(OH)D** **(nmol/L)**	**Vitamin D_3_** **Dose (IU)** **Treatment,** **Placebo,**	**Achieved 25(OH)D** **(nmol/L)**	**Variable**	**Baseline**	**Achieved**
Mohamad, 2016 [89]	100 T2DM patients	NA 47 ± 6	40 ± 13	4500/d 2 mo	124 ± 44	FBG (mg/dL)	F: 145 ± 29 M: 126 ± 21	F: 137 ± 28 * M: 121 ± 19 *
						HbA1c (%)	F: 7.7 ± 1.0 M: 8.1 ± 1.4	F: 7.3 ± 0.7 * M: 7.3 ± 1.0 *
Sluyter, 2017 [56]	256 [150 with 25(OH)D <50 nmol/L]	65 ± 9	38 ± 9	100,000/mo; 0 1.1 yr (0.9–1.5 yr)	96 ± 7	Aortic BP (mmHg)	72 ± 6	68.9 ± 5.5 −7.5 (−14.4 to −0.6) CtP
						Pulse wave velocity (m/s)	9.2 ± 1.8	8.9 ± 1.5 −0.3 (−0.6 to −0.1) CtP
						Peak reservoir pressure (mmHg)	125 ± 18	112 ± 12 −9 (−15 to −2) CtP
Mirhosseini, 2017 [57]	40 hypertensives not taking BP medications	27 *±* 6 NA	82 ± 38	4400 to 7800/d	113 ± 35	SBP (mmHg)	156 ± 15	138 ± 21 **
Imga, 2019 [90]	72 women	28 ± 1 41 ± 10	15 (7–40)	NA	87 (66–176)	LDL-C (mg/dL)	137 ± 38	128 ± 36 *
						FBG (mg/dL)	92 ± 6	87 ± 7 **
						HOMA-IR	3.6 (1.0–6.7)	2.0 (0.7–3.2) **
	50 women	36 ± 4 41 ± 10	14 (8–66)	NA	87 (67–142)	LDL-C (mg/dL)	132 ± 39	121 ± 33 *
						FBG (mg/dL)	92 ± 8	92 ± 6 **
						HOMA-IR	4.2 (2.8–10.8)	2.4 (1.2–7.0) **
[Li, 2021 [91]	1699 people receiving vitamin D supplements	NA	82 (58–110)	NA	+25 vs. −25	LDL-C (mg/dL)		−8 (−7 to −9) **
						TG (mg/dL)		−25 (−23 to −29) **
Pecoraro, 2022 [92]	58 children	31 ± 4 9–15	41 ± 16	100,000/mo	82 ± 42	LDL-C (mg/dL)	117 ± 12	102 ± 8 *p* = 0.005
						TG (mg/dL)	83 ± 35	80 ± 35 (NS)
Sabico, 2023 [93]	62 F	30 ± 4 39 ± 12	30 ± 12	50,000/wk for 2 mo; then twice a month, then 1000/d to end of 6 mo	66	HDL-C (mmol/L)	1.03 ± 0.4	+16% (*p* = 0.04)
	58 M	29 ± 4 43 ± 10	35 ± 10		71		0.98 ± 0.3	+7% (*p* = 0.001)

*, *p* < 0.05; **, *p* <0.001; CtP, compared to placebo; day; F, female; FBG, fasting blood glucose; HDL-C, high-density-lipoprotein-cholesterol; HOMA-IR, homeostasis model assessment of insuline risistance; M, male; mo, month; m/s, meters per second; NA, not available; NS, not significant; TG, triglycerides; wk, week.

**Table 4:** How Hill’s criteria for causality [32] are satisfied with vitamin D and CVD incidence.

Criterion	How Satisfied	References
Strength of association	Prospective cohort studies (Table 1)	[28]
Consistency	Prospective cohort studies (Table 1)	
Temporality	Prospective cohort studies (Table 1)	[28]
Biological gradient	Prospective cohort studies (Table 1)	
Coherence with known science	Vitamin D supplementation reduces CDRFs, and serum 25(OH)D concentrations are correlated inversely with CDRFs	[111]
Plausibility	Mechanisms	[97,98,99,110,112]
Experiment	Prospective observational studies of vitamin D supplementation	[67,68]
Analogy	The effect of vitamin D in reducing the risk of many other types of disease	[8]
Accounting for confounding factors [113]	Cold temperature, nitric oxide	[50]

CDRFs, cardiovascular disease risk factors.
